# The Polyphenol–Microbiota Axis: Molecular Mechanisms, Metabolic Pathways, and Therapeutic Perspectives in Human Health

**DOI:** 10.3390/jpm16030142

**Published:** 2026-03-02

**Authors:** Andrea Ballini, Simona Nicole Barile, Alfredo De Rosa, Maria Eleonora Bizzoca, Mariarosaria Boccellino, Salvatore Scacco, Stefania Cantore, Lorenzo Lo Muzio, Francesco Massimo Lasorsa, Roberto Arrigoni

**Affiliations:** 1 Department of Life Science, Health and Health Professions, Link Campus University, 00165 Rome, Italy; a.ballini@unilink.it (A.B.); a.derosa@unilink.it (A.D.R.); m.boccellino@unilink.it (M.B.); s.cantore@unilink.it (S.C.); 2 CNR Institute of Biomembranes, Bioenergetics and Molecular Biotechnologies (IBIOM), 70124 Bari, Italy; simonanicole.barile@ibiom.cnr.it (S.N.B.); francesco.massimo.lasorsa@uniba.it (F.M.L.); 3 Department of Clinical and Experimental Medicine, University of Foggia, 71122 Foggia, Italy; mariaeleonora.bizzoca@unifg.it (M.E.B.); lorenzo.lomuzio@unifg.it (L.L.M.); 4 School of Medicine, University of Bari Aldo Moro, 70124 Bari, Italy; 5 Department of Biosciences, Biotechnologies and Environment, University of Bari Aldo Moro, 70125 Bari, Italy

**Keywords:** polyphenols, gut microbiota, microbial metabolites, oxidative stress, AMPK, NF-κB, Nrf2, personalized nutrition, precision nutrition, chronic disease prevention, multi-omics

## Abstract

Polyphenols are a diverse class of bioactive phytochemicals increasingly recognized for their ability to modulate human physiology through extensive interactions with the gut microbiota. This review provides a comprehensive and updated synthesis of the bidirectional polyphenol–microbiota relationship, emphasizing how dietary polyphenols reshape microbial community structure while intestinal microorganisms metabolize polyphenols into smaller, more bioavailable derivatives. These microbial metabolites—such as urolithins, phenolic acids, and dihydroresveratrol—exert amplified biological activities compared to their parent molecules, acting on key molecular pathways linked to oxidative stress, inflammation, energy homeostasis, and metabolic regulation. Through integration of mechanistic studies, multi-omics analyses, and emerging clinical evidence, this review outlines the potential of the polyphenol–microbiota–metabolite axis as a target for precision nutrition and microbiota-informed therapeutic interventions. The manuscript highlights ongoing challenges, including inter-individual variability in polyphenol metabolism, and proposes future research directions to advance the field of personalized nutrition.

## 1. Introduction

Polyphenols constitute one of the most abundant and structurally diverse families of phytochemicals found in the human diet [1]. They are present in fruits, vegetables, tea, coffee, cocoa, wine, nuts, cereals, and spices. Over the past several decades, a wealth of epidemiological, clinical, and mechanistic studies has associated high polyphenol intake with a reduced risk of chronic diseases, including cardiovascular disease, metabolic syndrome, type 2 diabetes, neurodegenerative disorders, and certain cancers [2,3,4,5,6]. Although polyphenols have been historically described as antioxidants, their effects in vivo extend far beyond radical scavenging, encompassing anti-inflammatory, antimicrobial, metabolic, and signaling-modulatory activities.

A central factor influencing the biological activity of dietary polyphenols is their extensive interaction with the gut microbiota. The human gastrointestinal tract contains trillions of microorganisms that collectively encode far more genes than the human genome [7]. This microbial ecosystem plays essential roles in digestion, nutrient absorption, immune maturation, and neuroimmune communication [8]. Increasing evidence demonstrates that the gut microbiota and polyphenols engage in a highly dynamic and reciprocal relationship: polyphenols modulate microbial composition and activity, while microbes transform polyphenols into metabolites with enhanced physiological impact [9,10].

The apparent paradox of polyphenols’ low intestinal absorption and their widely documented systemic benefits can be resolved through understanding the gut microbiota’s essential role in polyphenol metabolism. In the colon, unabsorbed polyphenols undergo microbial biotransformation through hydrolysis, dehydroxylation, demethylation, and ring cleavage [10]. The resulting low-molecular-weight metabolites have improved bioavailability and often exhibit more potent biological effects. These metabolites influence host cellular responses by modulating signaling pathways related to oxidative stress, inflammation, and energy metabolism.

This review provides a detailed, integrated analysis of the polyphenol–microbiota axis, covering polyphenol classification and metabolism, microbial community responses, mechanistic insights into host–microbial signaling, and evidence from clinical trials. We also highlight emerging opportunities for microbiota-targeted nutrition interventions.

## 2. Polyphenols: Diversity, Sources, and Bioavailability

**Figure 1:**
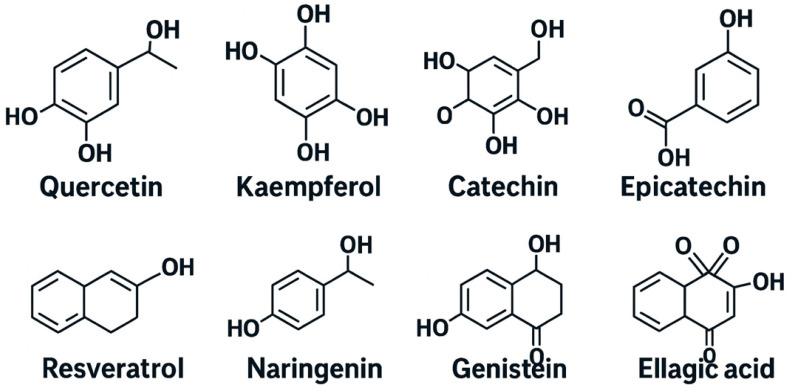
Chemical structure of different polyphenols.

Polyphenols comprise a broad and heterogeneous group of phytochemicals characterized by one or more aromatic rings bearing hydroxyl groups (Figure 1). They are traditionally classified into four principal categories—flavonoids, phenolic acids, stilbenes, and lignans—each encompassing numerous bioactive compounds found in everyday foods and beverages [11].

Flavonoids represent the largest and most extensively studied class, including subclasses such as flavonols (quercetin, kaempferol), flavan-3-ols (catechins, epicatechins), flavones (apigenin, luteolin), and anthocyanins (cyanidin, delphinidin). These compounds are abundant in fruits, tea, cocoa, red wine, and vegetables, and have been linked to cardiovascular protection and anti-inflammatory effects.

Phenolic acids, including hydroxybenzoic (e.g., gallic acid) and hydroxycinnamic (e.g., caffeic, ferulic) acids, occur mainly in coffee, grains, and berries. They contribute substantially to total dietary polyphenol intake and are recognized for their antioxidant and antimicrobial activities.

Stilbenes, exemplified by resveratrol from grapes and red wine, exhibit potent cardiometabolic and anticancer properties. Similarly, lignans, found in flaxseed, sesame, and whole grains, display estrogenic and antioxidant effects and may play a role in hormone-related disease prevention.

Despite their abundance, the bioavailability of polyphenols is relatively low, as only a small fraction (5–10%) is absorbed in the small intestine [12]. The remaining compounds reach the colon largely intact, where the gut microbiota catalyzes their biotransformation into smaller metabolites with enhanced absorption and biological activity [13,14].

The complexity of these transformations underscores the essential interplay between diet and microbiota. The diversity and metabolic capacity of an individual’s gut microbial community determine how efficiently polyphenols are converted into bioactive metabolites, influencing both inter-individual variability in responses and the therapeutic potential of polyphenol-rich diets.

Hence, polyphenols act as prebiotic-like compounds, reaching the colon where they serve as substrates for specific bacterial taxa. The resulting metabolites exhibit higher bio-efficacy and systemic availability than their precursors. Understanding this microbial conversion process provides a biochemical basis for designing personalized dietary interventions that optimize polyphenol metabolism and health outcomes.

## 3. The Gut Microbiota: Composition and Function

The human gut microbiota is a highly complex and dynamic ecosystem comprising trillions of microorganisms—including bacteria, archaea, fungi, and viruses—that collectively encode over 100 times more genes than the human genome [15]. This community exerts profound influences on host physiology [16], affecting nutrient metabolism [17], immune system maturation [18], and even neurobehavioral regulation [19].

**Figure 2:**
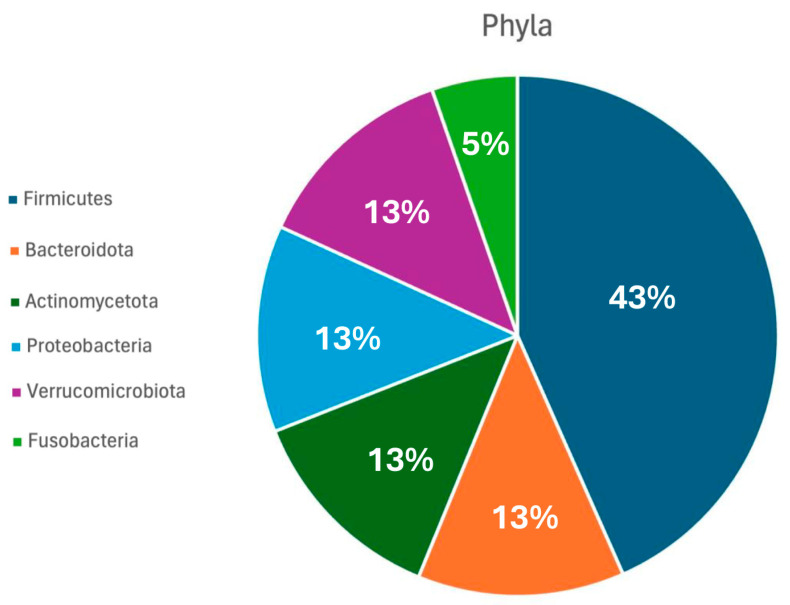
Gut microbiota composition. Diagram illustrating the phyla and their relative abundance in the human gut microbiota flora.

Four major bacterial phyla dominate the human gut: Firmicutes, Bacteroidetes, Actinobacteria, and Proteobacteria (Figure 2). Within these groups, genera such as *Bifidobacterium*, *Lactobacillus*, *Faecalibacterium*, and *Akkermansia* play essential roles in maintaining gut homeostasis. They contribute to the fermentation of complex polysaccharides, production of short-chain fatty acids (SCFAs)—notably acetate, propionate, and butyrate—and the synthesis of vitamins (e.g., vitamin K, B-group vitamins) [20].

SCFAs are particularly significant in linking microbial metabolism to host health. They provide an energy source for colonocytes, regulate gut pH, reinforce tight-junction integrity, and exert systemic anti-inflammatory effects through the inhibition of histone deacetylases and activation of G-protein-coupled receptors (GPR41, GPR43) [21]. These mechanisms collectively promote intestinal barrier function and immune homeostasis.

Dysregulation of the microbiota—termed dysbiosis—is associated with numerous pathologies, including inflammatory bowel disease, obesity, metabolic syndrome, and neurodegenerative disorders [22]. Thus, dietary modulation of microbiota composition represents a promising strategy for disease prevention and health promotion.

## 4. Mechanistic Interplay Between Polyphenols and Gut Microbiota

The relationship between polyphenols and gut microbiota is profoundly bidirectional and synergistic [23]. Experimental studies have demonstrated that polyphenol-rich diets increase microbial diversity, richness, and β-diversity, indicators of a stable and resilient gut ecosystem [24,25]. For instance, grape proanthocyanidins enhance the abundance of *Bifidobacterium* and *Akkermansia*, correlating with improved intestinal barrier function, lower intestinal permeability, and reduced systemic inflammation [26]. Similarly, catechins and quercetin modulate the microbiota by stimulating SCFA-producing bacteria and suppressing inflammatory Proteobacteria [27].

The mechanisms underlying these effects involve both direct antimicrobial actions and indirect ecological modulation. Polyphenols can disrupt bacterial membranes [28], inhibit quorum sensing [29], or deprive pathogens of essential nutrients such as iron [30], while simultaneously supporting beneficial commensals through metabolic cross-feeding and modulation of redox balance [31].

Mechanistically, polyphenols modulate the microbiota through antimicrobial effects, including inhibition of bacterial enzymes, which preferentially impact pathogenic species such as *Clostridium difficile* and *Escherichia coli* [32]. Additionally, polyphenols influence microbial metabolism by altering redox conditions and promoting cross-feeding networks that enhance SCFA production [31]. Collectively, these actions position polyphenols as prebiotic-like compounds capable of reshaping microbial communities and promoting a eubiotic state associated with metabolic, inflammatory, and immunological benefits [33].

**Figure 3:**
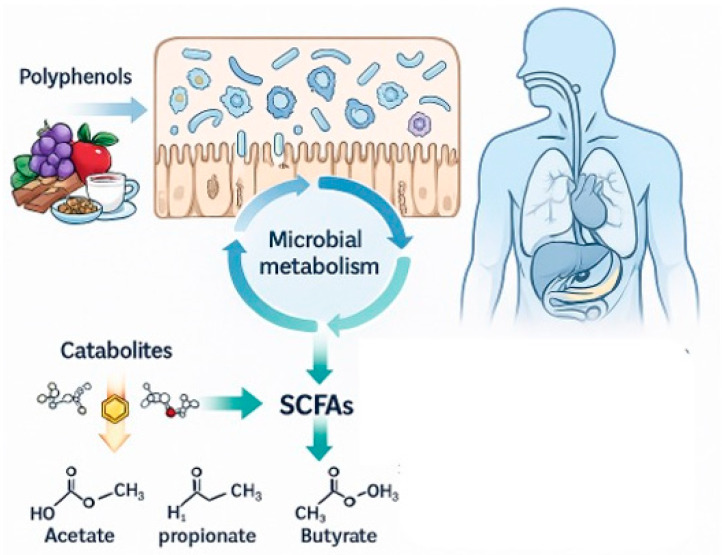
Microbiota Modulation by Dietary Polyphenols. Unabsorbed polyphenols reach the colon, selectively stimulate beneficial bacteria (*Bifidobacterium*, *Lactobacillus*, *Akkermansia*), inhibit pathogenic taxa (*Clostridium*, *E. coli*), and enhance production of short-chain fatty acids (acetate, propionate, butyrate).

Microbiota shifts induced by polyphenols have downstream impacts on metabolic, immune, and inflammatory pathways. Increased SCFA production enhances energy metabolism and insulin sensitivity, while reduced lipopolysaccharide (LPS) levels mitigate systemic inflammation [34]. Additionally, enrichment of *Akkermansia muciniphila*—a mucin-degrading bacterium linked to improved metabolic profiles—has been consistently observed in response to flavonoid and resveratrol intake [35]. For example, green tea catechins have been shown to enhance propionate-producing *Bacteroides* while reducing endotoxin-producing *Proteobacteria*, linking polyphenol intake to improved metabolic health (Figure 3) [36].

### Microbial Biotransformation of Polyphenols

The gut microbiota plays an essential role in unlocking the bioactivity of polyphenols through enzymatic conversion into low-molecular-weight metabolites. Microbial enzymes catalyze a range of reactions—hydrolysis, reduction, decarboxylation, demethylation, and ring cleavage—yielding metabolites with greater solubility and systemic availability. Only 5–10% of polyphenols are absorbed in the small intestine: the remainder undergo microbial metabolism in the colon to generate bioactive products. Table 1 highlights that the health effects attributed to dietary polyphenols are largely mediated by their microbial-derived metabolites rather than by the parent compounds themselves.

This underscores the pivotal role of the gut microbiota in shaping polyphenol bioactivity, clinical relevance, and interindividual variability in nutritional responses. In fact, following ingestion, most polyphenols reach the colon in an unmetabolized or partially metabolised form, where they are subjected to a wide range of microbiota-driven reactions. These processes markedly influence polyphenol bioavailability and biological activity.

Flavanols, together with proanthocyanidins, are primarily converted into phenolic acids and phenyl-γ-valerolactones, metabolites that have been linked to antioxidant and cardioprotective effects [45]. Flavonols, flavones, and flavanones mainly undergo deglycosylation and ring fission, yielding low-molecular-weight phenolic acids that contribute to anti-inflammatory and vasoprotective activities. Anthocyanins, which are inherently unstable in the gastrointestinal tract, are rapidly degraded into phenolic acids such as protocatechuic acid, supporting vascular health [46].

Isoflavones exemplify the strong interaction between diet and gut microbiota, as their microbial conversion into equol or O-desmethylangolensin leads to substantial interindividual variability in estrogenic and cardiometabolic responses [47]. Dietary lignans are metabolised into bioactive enterolignans, notably enterodiol and enterolactone, which are associated with hormonal modulation, antioxidant activity, and potential anticancer effects [48,49]. Stilbenes, including resveratrol, are transformed into dihydroresveratrol, a more stable metabolite with relevant cardioprotective and metabolic regulatory properties [50].

Curcuminoids are predominantly reduced and hydrogenated by gut microbiota, resulting in the formation of tetrahydrocurcumin, a metabolite with enhanced anti-inflammatory and neuroprotective potential [51]. Finally, ellagitannins, a subclass of hydrolysable tannins, are converted into urolithins, compounds extensively studied for their beneficial effects on gut health, mitochondrial function, and metabolic regulation [52].

This microbial transformation thus reconciles the apparent paradox between polyphenols’ poor absorption and their potent systemic effects. The microbiota effectively acts as a metabolic amplifier, converting dietary precursors into functional postbiotics that interact with host cells.

These microbiota-mediated effects extend beyond the gut, influencing the gut–liver [53], gut–brain [54], and gut–immune axes [55]. For example, microbial metabolites derived from polyphenols can cross the intestinal barrier and act on distant organs, contributing to cardiovascular protection, neuroprotection, and immune regulation [56].

## 5. Molecular Pathways and Host Systemic Effects

The bioactive metabolites generated through microbial transformation of polyphenols exert a wide range of regulatory actions on host physiology by targeting key molecular pathways involved in inflammation, oxidative stress, energy metabolism, cellular homeostasis, epithelial integrity and epigenetic remodeling.

### 5.1. Modulation of Nrf2

The Nrf2 (nuclear factor erythroid 2–related factor 2) –Keap1 axis is one of the primary targets of polyphenol-derived metabolites [57]. Compounds such as urolithin A, dihydroresveratrol, and phenyl-γ-valerolactones modify reactive cysteine residues on Keap1 or alter cellular redox tone, leading to the release of Nrf2 from the Keap1 complex, preventing the ubiquitin-mediated degradation, and nuclear accumulation and binding to ARE (antioxidant response elements) [58]. This induces a broad cytoprotective program encompassing antioxidant enzyme activity (HO-1, NQO1, SOD, GPx), phase II detoxification enzymes, glutathione synthesis, and mitochondrial biogenesis through crosstalk with PGC1-α [59].

Nrf2 activation also indirectly suppresses inflammatory signaling by reducing ROS-dependent NF-κB activation and supports redox balance, mitochondrial integrity, and epithelial resilience, particularly within the gastrointestinal tract [60].

### 5.2. Modulation of NF-κB and the Inflammatory Cascade

Polyphenol metabolites attenuate chronic inflammation by interfering with NF-κB (nuclear factor-kappa B) signaling cascade at multiple regulatory checkpoints: (a) inhibition of IKK complex activity [61], preventing IκBα phosphorylation and degradation, lowering the expression of pro-inflammatory mediators (TNF-α, IL-6, IL-1β, COX-2) [62,63]; (b) reduction in ROS-mediated NF-κB activation, due to improved antioxidant buffering [64]; (c) epigenetic downregulation of pro-inflammatory genes through HDAC inhibition and altered histone acetylation patterns [65]; (d) suppression of TLR4 signaling, following reduced LPS translocation associated with improved gut barrier function [66].

Downstream consequences include lowered transcription of pro-inflammatory cytokines (TNF-α, IL-6, IL-1β), chemokines (MCP-1), and enzymes such as COX-2 and iNOS [67]. This contributes to systemic reductions in low-grade inflammation central to obesity, cardiometabolic disease, and gut inflammatory disorders.

### 5.3. AMPK Activation and Metabolic Reprogramming

A third fundamental target is AMP-activated protein kinase (AMPK), a master regulator of cellular energy homeostasis. Several microbial metabolites activate AMPK in metabolic tissues, enhancing fatty acid oxidation, improving glucose uptake, increasing mitochondrial biogenesis, and reducing hepatic lipogenesis [68]. Through these effects, the polyphenol–microbiota axis influences body weight regulation, insulin sensitivity, and lipid metabolism. Microbial metabolites dihydroresveratrol and phenyl-γ-valerolactones, regulate energy homeostasis through activation of AMP-activated protein kinase (AMPK). The mechanisms of action include:

- increased AMP/ATP ratio via mitochondrial hormesis;
- direct phosphorylation of AMPKα subunit by upstream kinases (LKB1, CaMKKβ) [69];
- improved mitochondrial efficiency and reduced oxidative burden, reducing ATP waste [70].

AMPK activation orchestrates a global metabolic shift: it stimulates fatty acid oxidation via ACC inhibition [71]; enhances glucose uptake through GLUT4 translocation [72]; reduce hepatic gluconeogenesis [73]; inhibits lipogenesis via downregulation of SREBP-1c [74]; and stimulates autophagy through ULK1 activation [75].

This pathway links microbial metabolism of polyphenols to improved insulin sensitivity and metabolic health.

### 5.4. Endothelial and Immune Signaling

Endothelial and immune modulation are also another target of the polyphenol–microbiota interaction: phenolic metabolites enhance nitric oxide synthase (eNOS) activity, improving vascular tone and reducing oxidative LDL formation, while also regulating T-cell and macrophage activity through redox signaling. Polyphenol metabolites also modulate eNOS activation, increasing nitric oxide levels, leading to improved vascular function [76]; in addition, their action on tight junction expression (ZO-1, occludin, which is the target of urolithins) enhanced epithelial integrity [77]. It was demonstrated that pomegranate polyphenols directly suppress macrophage inflammatory responses and promote M1 to M2 switch in macrophages through redox- and AMPK-mediated mechanisms, leading to immune cell polarization [78]. Gut phenolic metabolites, particularly phenolic acids, can modulate AhR (aryl hydrocarbon receptor) activation, influencing immune tolerance and barrier repair [79].

### 5.5. Epigenetic and Mitochondrial Mechanisms

Emerging evidence shows that metabolites such as urolithin A are able to regulate mitochondrial quality control via mitophagy activation (PINK1/Parkin) [80], chromatin remodeling through inhibition of class I/II histone deacetylases (HDACs) [81], and microRNA expression associated with inflammatory and metabolic regulation [82]. These epigenetic and mitochondrial effects provide a durable link between diet, microbiota metabolism, and host phenotypes.

**Figure 4:**
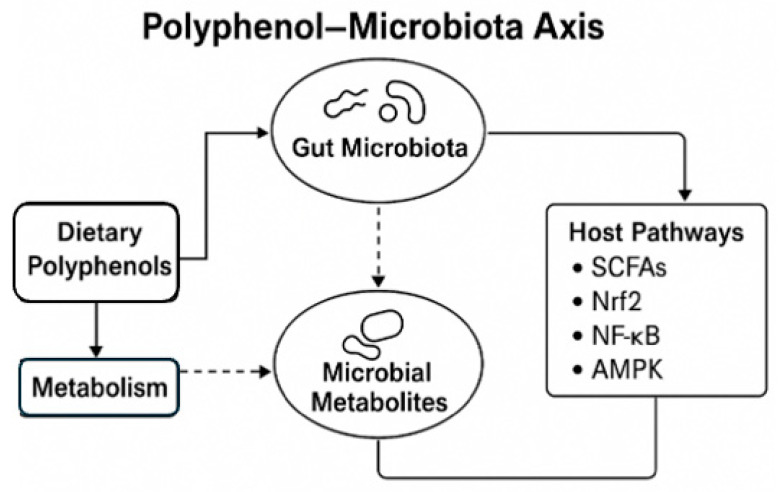
Molecular Pathways Activated by Polyphenol-Derived Metabolites. Integrated schematic illustrating how gut microbiota converts polyphenols into metabolites (urolithins, dihydroresveratrol, tetrahydrocurcumin) that activate Nrf2, inhibit NF-κB, and stimulate AMPK pathways. Downstream effects include reduced oxidative stress, improved lipid/glucose metabolism, and suppression of chronic inflammation.

The combined activation of Nrf2, inhibition of NF-κB, and metabolic reprogramming via AMPK, together with endothelial, immune, and epigenetic modulation, forms a multi-layered mechanistic framework explaining the broad systemic benefits of polyphenol-rich diets (Figure 4). This network demonstrates how microbial metabolism transforms poorly absorbed dietary compounds into potent signaling molecules that influence host health across multiple organs and systems.

Together, these pathways establish the polyphenol–microbiota–host axis as a critical determinant of systemic antioxidant capacity, immune balance, and metabolic homeostasis. The mechanistic interplay between polyphenols and the gut microbiota represents a symbiotic biochemical network that sustains host health (Figure 4). Microbial diversity ensures efficient polyphenol metabolism, while dietary polyphenols act as microbial modulators capable of reshaping the intestinal ecosystem toward a eubiotic state that favors host health—forming a reciprocal feedback loop essential for metabolic, cardiovascular, and immune regulation.

## 6. Dietary Influence, Clinical Evidence, and Translational Implications

Diet plays a central role in shaping both polyphenol availability and the composition of the gut microbiota, making dietary patterns a key determinant of the polyphenol–microbiota axis [83]. Diets rich in fruits, vegetables, tea, coffee, red wine, and whole grains provide abundant sources of polyphenols, which interact with the intestinal microbiota to generate beneficial metabolites [84]. Conversely, Western-style diets—high in saturated fats and refined carbohydrates—are typically low in polyphenols and associated with dysbiosis, inflammation, and metabolic dysfunction [85,86]. Regular consumption of polyphenol-rich diets, such as the Mediterranean diet, promotes microbial diversity and enriches beneficial taxa like *Bifidobacterium*, *Lactobacillus*, and *Akkermansia muciniphila* [87]. These shifts correlate with increased production of SCFAs, improved mucosal barrier integrity, and reduced circulating inflammatory markers.

Emerging evidence suggests that synergistic effects between polyphenols and dietary fiber enhance these outcomes: fibers provide fermentable substrates, while polyphenols modulate microbial composition, together fostering a resilient, health-promoting microbiota [88].

Clinical trials investigating polyphenols, gut microbiota, and disease prevention have begun to reveal promising insights into the therapeutic potential of polyphenol-rich diets in managing and preventing chronic diseases [89,90]. However, given the complexity of polyphenol–microbiota interactions, rigorous, large-scale clinical studies are still needed to fully understand these effects [91]. Recent trials have explored the impact of specific polyphenols, such as resveratrol, curcumin, and catechins, on metabolic health, inflammatory conditions, and gut microbiota composition [92,93].

The use of resveratrol has shown improvements in insulin sensitivity and reductions in inflammatory markers in individuals with type 2 diabetes, outcomes that are linked to favorable shifts in gut microbiota composition, including increased levels of beneficial bacteria like *Akkermansia muciniphila* [94]. Similarly, clinical studies examining the effects of curcumin in inflammatory bowel disease (IBD) have reported reductions in gut inflammation and symptomatic relief, likely due to curcumin’s impact on the microbiota and its anti-inflammatory properties [93]. Catechins, especially from green tea, have also been investigated in clinical trials for their potential to lower cholesterol levels and blood pressure, with results suggesting that these effects are mediated in part by the microbiota’s transformation of catechins into bioactive metabolites that promote cardiovascular health [95].

Clinical evidence also supports the role of polyphenols in modulating gut microbiota. In a randomized, double-blind, placebo-controlled trial involving healthy obese adults, the effects of a polyphenol-rich cranberry beverage on intestinal permeability and gut microbiota were examined. After two weeks of treatment, the abundance of *Faecalibacterium prausnitzii* and *Eggerthella lenta* increased, while *Bifidobacterium* spp. decreased [96]. These findings demonstrate that polyphenols can alter gut microbiota composition in humans. Supplementation with resveratrol, anthocyanin-rich berries, or grape polyphenols increased insulin sensitivity, lowered fasting glucose, and modulated gut microbiota toward a higher Bacteroidetes/Firmicutes ratio.

Trials on polyphenol-rich diets, such as the Mediterranean diet, have additionally shown significant reductions in cardiovascular and cancer risks, partly attributed to the high intake of polyphenols and their positive modulation of the gut microbiota [97]. Intervention studies with green tea catechins and cocoa flavanols have shown improved endothelial function and reduced blood pressure, associated with elevated levels of *Akkermansia muciniphila* and enhanced NO-mediated vasodilation [98]. Curcumin and quercetin supplementation have reduced intestinal inflammation, improved gut permeability, and increased beneficial Lactobacillus species in the treatment of inflammatory bowel disease (IBD) [99]. Polyphenol metabolites (e.g., urolithin A, tetrahydrocurcumin) attenuated markers of oxidative stress and inflammation in patients with mild cognitive impairment, implicating gut–brain axis modulation, suggesting a neuroprotective effect. Two different clinical studies in healthy subjects have shown that supplementation with 7 targeted probiotic strains plus leaf extract of *Lagerstroemia speciosa* improved glycemic control [100], and by using probiotics in combination with prebiotic fructo-oligosaccharides (FOS) and Echinacea improved stress response and inflammation markers [101].

Nevertheless, the efficacy of prebiotic and probiotic supplementation in routine clinical settings has not yet been definitively established. Although the available evidence appears encouraging, the clinical studies discussed above frequently yield inconclusive and, at times, conflicting results. The European Prospective Investigation into Cancer and Nutrition (EPIC) study, which included nearly half a million participants from 10 European countries, reported an inverse association between polyphenol intake and colon cancer risk in men [102]. However, the same study found no association between dietary polyphenol intake and the risk of differentiated thyroid cancer or overall colorectal cancer [102]. In contrast, a positive association between polyphenol intake and rectal cancer was observed in women [102]. Such variability may be attributed to multiple factors, including limitations in study design, insufficient sample sizes, and the possibility that observed associations reflect simple correlations rather than causal effects.

A search of the clinicaltrials.gov database using the words “prebiotics and probiotics” reveals 252 studies; however, only 4% (11 studies) reported any results [103]. Expanding the search to only the terms “prebiotic” or “probiotic”, we find 637 and 2636 clinical studies, respectively, but the percentage of studies that led to results remains substantially unchanged [104,105]. In the absence of more robust experimental and clinical evidence, supplementation may represent a supportive intervention; however, it should be integrated within a personalized therapeutic strategy rather than regarded as a universal alternative to conventional pharmacological treatments.

These clinical trials highlight the importance of understanding individual differences in microbiota composition and the need for personalized approaches in future studies. Researchers are increasingly focusing on the potential of microbiota-targeted therapies, such as probiotics or prebiotics, in conjunction with polyphenol-rich diets, to enhance treatment outcomes [106]. Ongoing and future clinical trials will be essential in establishing standardized guidelines for polyphenol intake, determining optimal dosages, and clarifying the long-term health benefits of polyphenol–microbiota interactions in disease prevention [91].

### Translational Implications: Toward Personalized and Microbiota-Targeted Nutrition

The growing body of clinical and omics-based data underscores the potential of polyphenol-rich diets as microbiota-targeted therapies. However, substantial inter-individual variability in microbial composition leads to differences in metabolite production and therapeutic efficacy. These differences support the emerging field of precision nutrition, where polyphenol intake and combinations can be optimized based on a person’s microbiome signature. Metagenomic and metabolomic profiling may soon enable the prediction of individual responses to specific polyphenol interventions, facilitating personalized dietary recommendations for chronic disease prevention.

Future strategies may combine novel microbiota-targeted approaches—such as co-administration of probiotics, prebiotics, or postbiotics—may enhance polyphenol metabolism and bioefficacy, opening new frontiers in nutritional therapeutics. For instance, administering *Bifidobacterium adolescentis* alongside ellagitannin-rich foods could increase urolithin production [107], whereas supplementation with butyrate-producing bacteria may potentiate SCFA-mediated benefits [108].

However, the clinical translation of polyphenols-microbiota coadministration is not an easy task: many existing trials are short-term or small-scale, with short follow-up periods, heterogeneous outcomes, and limited external validation. Large, controlled studies with standardized polyphenol doses and well-characterized microbiota endpoints are required to establish causality and inform evidence-based dietary recommendations. At the same time, multi-omics analyses and continuous digital monitoring remain costly and operationally demanding, while many clinicians lack training to interpret omics-based outputs. Targeting clinician education is mandatory to ensure that digital health and personalized nutrition recommendations are interpretable, actionable, and consistent with routine care.

Clinical translation will further depend on demonstrating clear added value by using clinically meaningful and measurable endpoints, supported by external validation prior to adoption. Consensus guidelines emphasize that implementation should favor clinician-oriented decision support that delivers interpretable, guideline-aligned recommendations with explicit uncertainty, rather than exhaustive taxonomic outputs.

In this regard, Artificial Intelligence (AI) is an increasingly prominent technology in healthcare, with significant potential to reshape clinical nutrition. It can support the analysis of complex datasets and the delivery of personalized nutritional interventions. AI may help clinicians make more informed decisions about nutritional needs, disease prevention, and treatment strategies [109]. By processing large volumes of data, AI algorithms can uncover new relationships between dietary patterns and health outcomes, supporting evidence-based nutritional guidance. In addition, AI-driven tools and applications can help monitor dietary intake and offer real-time feedback. Despite these advantages, the use of AI in clinical nutrition raises ethical and regulatory issues, including concerns about data privacy and algorithmic bias. Further research is required to evaluate the effectiveness and safety of AI-based nutritional interventions. Overall, while AI holds considerable promise for advancing clinical nutrition, its implementation in practice must be carefully supervised to ensure patient safety and positive outcomes. Artificial intelligence could be an incredible tool to analyze big data and find consistent and useful clinical therapies.

Finally, feasibility, privacy, and equity must be integral to system design. The strong geographic bias of public microbiome datasets—heavily skewed toward Western, high-income populations—limits generalizability and risks exacerbating health disparities if tools are deployed without validation in diverse populations. At the same time, the convergence of microbiome profiling with sensitive omics and digital phenotypes heightens governance and privacy concerns, particularly in commercial or cross-institutional contexts.

In summary, clinical and translational studies collectively demonstrate that polyphenols act as dietary modulators of the gut microbiome, influencing human health through metabolite-mediated signaling. Harnessing this interplay through personalized nutritional strategies offers a promising route for preventing and managing chronic diseases.

## 7. Discussions and Future Perspectives

Polyphenols represent one of the most promising classes of bioactive dietary compounds, exerting wide-ranging effects on human health through their reciprocal relationship with the gut microbiota. The polyphenol–microbiota–metabolite axis functions as a dynamic system that translates dietary inputs into molecular and metabolic signals governing oxidative balance, immune modulation, and metabolic homeostasis. The interaction between polyphenols and intestinal microbes constitutes a bidirectional feedback loop: polyphenols shape microbial composition toward a eubiotic state, while the microbiota metabolizes them into bioactive derivatives with amplified physiological effects. This interdependence provides a mechanistic explanation for how dietary diversity and polyphenol intake can modulate systemic health and prevent chronic disease, bridging nutrition, microbiology, and molecular physiology.

Despite substantial progress, several challenges remain before these findings can be fully translated into dietary guidelines or therapeutic strategies: firstly, the inter-individual variability has to be considered. Differences in microbiota composition result in heterogeneous metabolic capacities for polyphenol biotransformation. Identifying microbial biomarkers predictive of high or low metabolite production (e.g., urolithin A producers) is essential for designing personalized interventions [110].

The intricate signaling networks linking microbial metabolites to host molecular targets still remain incompletely mapped. Advanced multi-omics integration combining metagenomics, metabolomics, transcriptomics, and proteomics will be crucial for elucidating these pathways.

The convergence of nutritional science, microbiome research, and data-driven omics heralds the emergence of precision nutrition—a paradigm in which dietary recommendations are tailored to individual microbial and metabolic profiles [111]. By mapping how distinct microbiota configurations influence polyphenol metabolism, clinicians and nutritionists will be able to customize dietary interventions to maximize therapeutic benefit [112].

**Figure 5:**
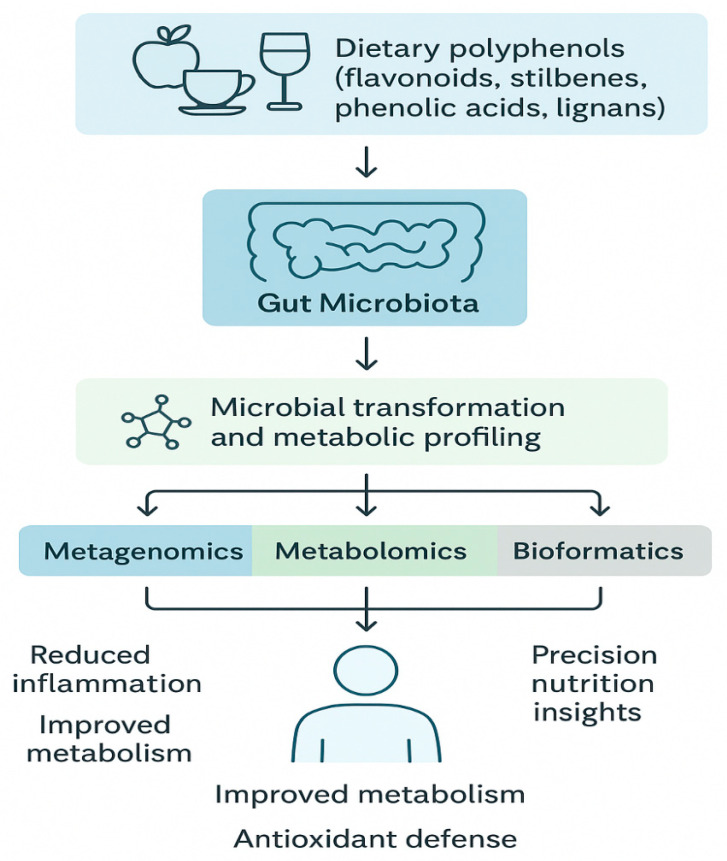
Future Perspectives: Integrative Framework for Polyphenol–Microbiota–Health Research. A conceptual model illustrating the transition from basic mechanistic understanding to personalized interventions. Highlights multi-omics tools (metagenomics, metabolomics, transcriptomics), AI-based data integration, and translation into individualized nutritional guidelines.

Finally, integrated multi-omics approaches connect microbial composition, metabolite profiles, and host physiological responses, enabling causal inference and the development of predictive models of polyphenol responsiveness [113]. This systems-level perspective supports the advancement of precision nutrition, allowing tailored dietary interventions based on an individual’s microbiome and metabolic signatures. By bridging microbial ecology, molecular mechanisms, and clinical phenotypes, *omics* technologies offer a powerful framework for understanding and optimizing the health benefits associated with polyphenol–microbiota interactions (Figure 5) [114].

Future research integrating artificial intelligence and personalized metabolomics will enable predictive models linking dietary polyphenol intake to measurable health outcomes; such approaches could redefine dietary management of chronic diseases through microbiome-informed precision nutrition strategies. Advances in multi-omics technologies—including metagenomics, metabolomics, and transcriptomics—allow for precise mapping of polyphenol-derived metabolites and their symbiotic interactions with the host [115,116]. Furthermore, inter-individual differences in microbiota composition introduce substantial variability in polyphenol metabolism, underscoring the importance of personalized nutrition strategies aimed at optimizing health outcomes.

## Figures and Tables

**Table 1:** Major classes of polyphenols, microbial transformations, representative metabolites, and main health effects.

Polyphenol Class	Principal Microbial Transformations	Representative Metabolites
Flavonols	Deglycosylation, dehydroxylation, ring fission [37]	Phenolic acids, hydroxyphenylacetic acids
Flavones	Deglycosylation, demethylation [38]	Phenolic acids
Flavanones	Deglycosylation, dihydroxylation [39]	Phenylpropionic acids
Isoflavones	Deglycosylation, reduction [40]	Equol, O-desmethylangolensin
Anthocyanins	Deglycosylation, ring cleavage [41]	Protocatechuic acid, phenolic acids
Proanthocyanidins	Depolymerisation, ring fission [42]	Phenyl-γ-valerolactones
Tannins (condensed & hydrolysable)	Hydrolysis, decarboxylation [43]	Gallic acid, urolithins
Chalcones	Reduction, isomerization [44]	Phenolic acids
Stilbenes	Hydrogenation, dihydroxylation [44]	Dihydroresveratrol
Lignans	Demethylation, dehydroxylation, dehydrogenation [43]	Enterolactone, enterodiol
Curcuminoids	Demethylation, hydroxylation, reduction and acetylation [42]	Tetrahydrocurcumin, dihydrocurcumin

## Data Availability

No new data were created or analyzed in this study.
